# Automated skin cancer detection using MedFusionNet with attention-based fusion of ConvNeXt and vision transformer

**DOI:** 10.1038/s41598-025-31816-2

**Published:** 2025-12-12

**Authors:** Muhammad Ahtsam Naeem, Shangming Yang, Muhammad Asim Saleem, Iram Javed, Ashir Javeed

**Affiliations:** 1 School of Information and Software Engineering, University of Electronic Science and Technology of China, Chengdu, China; 2 Department of Electrical Engineering, Center of Excellence in Artificial Intelligence, Machine Learning, and Smart Grid Technology, Faculty of Engineering, Chulalongkorn University, Bangkok, Thailand; 3 Department of Computer Science, Blekinge Institute of Technology, Karlskrona, Sweden

**Keywords:** Skin cancer classification, Deep learning, ConvNeXt, Vision Transformer, Attention mechanism, Cancer, Cancer imaging

## Abstract

Skin cancer, especially melanoma, the most severe type, has increased in recent decades. It develops from cells that grow abnormally and can invade the surrounding tissue and spread throughout the body. Early and accurate diagnosis is essential to prevent disease progression and allow for less invasive clinical treatment. The extraction of complex dermoscopic images and the improvement of lesion classification performance have significantly improved skin cancer diagnosis through the use of convolutional neural networks (CNNs). In this study, a novel deep convolutional neural network that combines ConvNeXt and Vision Transformer (ViT) architectures through an adaptive attention-based approach for advanced feature fusion to automatically multi-classify skin cancer samples. This model is evaluated on two dermoscopy benchmark datasets, including ISIC-2019 and HAM10000 and both datasets reflect the real-world problem of class imbalance. The evaluation results of MedFusionNet are calculated using various evaluation metrics, including accuracy, precision, recall and AUC and compared with deep learning algorithms such as ResNet50, MobileNet V2, DenseNet121 and ViT-B16. The experimental results show that MedFusionNet outperforms the current models with a classification accuracy of 98.80% and 97.90% for HAM10000 and ISIC-2019, respectively. Grad-CAM visualizations qualitatively show that the model focuses on clinically relevant lesion regions, providing interpretive insight without claiming complete causal explainability. The results show that the proposed model can efficiently handle multi-class tasks in dermatological imaging and MedFusionNet is a suitable choice for implementation in real-world computer-aided diagnosis systems.

## Introduction

Melanoma, the most malignant skin cancer, is a global health challenge due to its rapid progression, high metastatic potential and high mortality rate^1–3^. Among the various types of skin cancer, melanoma is of particular concern as it quickly becomes malignant and is fatal if detected too late. Early detection of melanoma significantly improves the chances of cure, which makes it clear why early diagnosis is so important^4,5^. Dermatologists are crucial in the use of classical diagnostic methods, but recognising a harmless mole and a dangerous melanoma at an early stage can be difficult for the inexperienced^6,7^. For this reason, better models are needed to detect infections quickly and accurately.

Artificial intelligence, especially deep learning (DL) and machine learning (ML), has now proven that it can make a big difference in medical diagnostics. CNNs^8^, which are part of DL, often provide strong results in the classification of images, including those from the medical field^9,10^. Numerous review studies confirm that DL-based systems have shown strong performance in various areas of medical diagnostics.^11^ describes how deep learning models excel at tasks such as disease detection, localization and segmentation in radiological and pathological images. In addition, meta-analyses by^12^ report that CNN-based medical classifiers often achieve diagnostic accuracy comparable to human experts, with pooled AUC values of over 0.90 in imaging tasks such as breast screening, ophthalmology and dermatology^13^. In the context of dermatology, several reviews and comparative studies emphasise the potential of CNNs and AI tools for the automatic classification of skin lesions^14–17^. These results emphasise the overall effectiveness and clinical potential of CNNs in image-driven diagnosis^18,19^. These models are good at recognising different patterns in images, which makes them useful for the classification of skin lesions^20,21^. Despite their achievements, the main problems of balancing the classes, dealing with noisy data and the generalisability of the models are still major problem.

In this study, we propose a novel deep learning model to address these problems and improve the accuracy of skin cancer classification. The proposed model integrates two powerful architectures, ConvNeXt and Vision Transformer (ViT), with an attention mechanism to leverage the strengths of both the convolutional architecture and transformation architecture^22,23^. The model can focus on important information by the way the attention mechanism selects and weights certain backbone features. With this design, the model performs significantly better on key evaluation metrics such as accuracy, recall, precision and area under the curve (AUC).

The model was tested using two different data sets based on clinical images of skin lesions. The experimental results show that the new transfer learning model performs better than VGG16, ResNet and MobileNetV2 in all aspects of the evaluation. Moreover, the model overcomes a common problem in medical imaging datasets, class imbalance, which makes it trustworthy in cases where the minority class is involved. This suggests that similar architectural strategies could potentially be adapted for other medical imaging tasks in future research.

The study contributes to the literature on the growing field of healthcare in general and oncology and dermatology in particular, where AI machines play an important role. The proposed model would improve the early diagnosis and the high accuracy of skin cancer. Future work could explore the adaptation of MedFusionNet to other areas of medical imaging, such as breast histology or chest X-rays, to assess the generalisability of the model to different diagnostic tasks. Additional explanatory measures such as gradient flow visualization, attention weighting heat maps and class activation maps (CAM) further increase the interpretability of the model and encourage healthcare professionals to accept the results.

The main contributions of this study are as follows:

(i) Integration of CNNs and ViTs in a novel model leads to better skin cancer classification.
(ii) The model was evaluated using the ISIC-2019, HAM10000 data and showed better results than the existing models.
(iii) The MedFusionNet architecture is modular and flexible by design, indicating potential adaptability to other medical imaging tasks, which will be explored in future studies.

This paper is organized as follows: Section Related work presents the main themes of the existing literature. Section Methods outlines the methods used in this study, including the preparation of the dataset and the model architecture. Section MedFusionNet: A ConvNeXt-ViT framework for skin cancer classification explains the proposed deep learning hybrid model. Section Experimental results and discussion presents the results of the experiments and Section Conclusion and future work concludes and gives an outlook for the future.

In this study, a hybrid deep learning method for skin cancer classification is presented, which can help in early detection and diagnosis. The results show that AI has the potential to both develop diagnostics and improve healthcare worldwide.

## Related work

Skin cancer is the most common of all cancers and can be fatal, so it is very important to detect it at an early stage. Since the development of deep learning methods, many researchers have focused on the use of CNNs for the automatic detection and classification of skin lesions. Several researchers have proposed new techniques to make the diagnosis of skin cancer more accurate and efficient. An important contribution was made by Shah et al.^24,25^, a comprehensive research study in which they investigated CNNs and ANNs for skin cancer diagnosis and concluded that deep learning techniques significantly improve performance. In addition, Nasreen et al.^23,26^ performed a comparison of current methods for skin image segmentation and particularly emphasised the use of CNNs for skin lesion classification and segmentation. The authors tested different CNN structures and proved that they work well in the identification of skin cancer.

A hybrid CNN model, which they presented in^27^, achieved 95% accuracy in classifying skin cancer in the HAM10000 dataset. The researchers found that their model performed better than other popular transfer learning models, VGG16 and InceptionV3. Optimising the ResNet model with the Whale optimisation algorithm, as performed by Tlaisun et al^3^, resulted in a 92% classification success rate for melanoma detection from the HAM10000 dataset. A further increase in model performance was observed when^28^ was implemented. Compared to AlexNet and VGG-16, the model achieved the best accuracy of 91.43 percent. This type of testing demonstrated the potential of DCNNs in detecting benign and malignant skin lesions. Keerthana et al.^29^ combined CNNs and machine learning and achieved an accuracy of 88.02% on the ISBI 2016 dataset. The two CNN models, DenseNet-201 and MobileNet, were used to extract image features, which were then classified using a Support Vector Machine (SVM).

Xu et al.^30^ proposed a system using a median filter, CNN for segmentation and Satin Bowerbird optimization for feature selection. With this model, they achieved 95% accuracy in detecting skin cancer, confirming the positive effect of preprocessing the data. Suganthi et al.^31^ also used ResNet50, added focal losses and used class weighting to solve the problem of skewed data. The model achieved a classification accuracy of 93.00% when tested with HAM10000. According to Popescu et al.^32^, a collective intelligence system based on different CNN structures was developed to recognise skin lesions. Their ensemble method with the different trained models of the HAM10000 set resulted in an average of 3% improved accuracy compared to the individual models. Such an approach illustrates that ensemble learning has the potential to enable reliable and accurate systems for skin cancer. The researchers use both technologies, CNN and SVM, to detect skin cancer. The researchers found that the CNN model was able to correctly classify the data with an accuracy of 91%, which is higher than the 86.6% result achieved by the SVM model. Similarly, Reis and Turk^33^ MABSCNET, a combination of depth-separable CNNs and a lightweight vision transformer and ensemble learning, which also achieved an accuracy of 92.74% on the ISIC-2020 dataset, indicating the success of combining CNNs and transformers in the early detection of skin lesions. This is consistent with the larger trend of fusion models, including that of Reis and Turk (2024)^34^, which used a fusion of CNN features and transformer-based attention to improve melanoma detector performance on the ISIC 2020 and other datasets.

Albahar in^35^ presented the framework for using CNN to classify skin conditions as well as a new regularizer for the standard deviation of filter weights. Their regularization method, based on the ISCIC 2018 dataset, achieved an accuracy of 97.49 percent.

DeeplabViT, the system developed by Ahmad et al.,^36^ an example of a hybrid deep learning architecture with DeepLabv3+ as a component for segmentation and a Vision Transformer (ViT) for classification of a skin lesion. Their dual-stage system is used to solve common problems in dermoscopic analysis, which include jagged lesion boundaries, low contrast and complicated lesion textures. DeepLabv3+ consisted of 9 convolutional blocks specialized for lesion segmentation and the ViT model implemented a patch token of size 7x7 and long-range dependency better than CNNs. Their model achieved the best accuracy on a number of datasets, e.g., 96.97% on ISIC-2019 and 100% on HAM10000. This demonstrates the increasing trend towards using CNN-transformer hybrid models in dermatological tasks, as well as the importance of benchmark comparisons in current studies. Similarly, Nazari and Garcia^37^ presented a resource-efficient attention-based model for automated melanoma diagnosis and showed that smaller attention-centered models with sufficient training can be quite competitive and thus resource-efficient. In addition, Dong et al.^38^ take advantage of multimodal learning by fusing the dermoscopic images and clinical metadata for segmentation and classification. Their results highlight the importance of using patient-specific information in the context of each diagnosis, which should be part of future research.

Zhao et al.^39^ presented a new system for detecting skin lesions using StyleGAN and DenseNet201. On the ISIC-2019 dataset, the selected method improved the classification performance by achieving a balanced multiclass accuracy of 93.64%. StyleGAN improved the quality of skin lesion images during classification when data enrichments were used. Viknesh et al.^40^, their model uses CNN to classify skin lesions and compares models such as AlexNet, LeNet and VGG-16. In both the mobile and web application, the CNN model is used, which achieved a classification rate of 91%, making it easier for dermatologists to identify skin lesions. According to Kondaveeti and Edupuganti^28^, their CNN approach to skin cancer detection was able to accurately classify images at a rate of 99%. Ali et al.^41^ presented a novel DCCN model for skin lesion classification. In a test with the HAM10000 dataset, their model outperformed many transfer learning models and achieved an accuracy of 91.43%.

Table 1 shows the summary of the empirical literature gap analysis. In recent years, the use of deep learning methods, particularly CNNs, has led to advances in the detection of skin cancer. This research shows that these models can streamline skin cancer diagnosis and increase the accuracy of decisions made by dermatologists. Beyond architectural innovations, recent research shows that hybrid loss functions such as combinations of cross-entropy, dice and focal losses can improve the robustness of the model, especially in medical imaging tasks^42,43^.

Recently, different fusion strategies combining convolutional neural networks (CNN) and attention mechanisms have been explored for the classification of skin lesions. The simple feature concatenation^44^ merges the results without adaptivity. Gating mechanisms^45^ provide weighted control over features, while cross-attention modules^46^ align spatial-semantic cues across backbones or with transformers. Although such approaches are effective, they often lead to additional complexity or require more computational resources. Our proposed softmax-based adaptive fusion strategy offers a simpler alternative that enables dynamic feature weighting with minimal architectural effort.

In addition, CNN-transformer hybrids have shown promising results in the detection of a wide range of cancers outside dermatology. Recent studies have applied such models to tasks such as the classification of breast cancer based on histopathology images^47^, the detection of cervical cancer based on smears^48^, the segmentation of colorectal polyps^49^ and the detection of gastrointestinal lesions using transformers and ensemble frameworks^50,51^. More comprehensive reviews also support this trend, highlighting that CNN transformer architectures offer the best performance in various cancer diagnoses, including breast, glioma and cervical cancer^52,53^. These results highlight the adaptability and effectiveness of hybrid deep learning models and justify the exploration of MedFusionNet for broader medical imaging applications.

**Table 1:** Summary of empirical literature gap analysis.

References	Year	Dataset	Contribution	Limitations
Shah et al.^24^	2023	HAM10000	A comprehensive study on the use of ANN and CNN for skin cancer detection.	Relatively limited to ANN and CNN models.
Nasreen et al.^26^	2022	Various data sets	Comparative study of state-of-the-art CNN-driven approaches for skin image segmentation.	Limited generalization due to specific image segmentation methods.
Ali et al.^27^	2023	HAM10000	DCNN model outperforms other transfer learning models with an accuracy of 91.43%.	Limited to binary classification (benign vs malignant).
Tlaisun et al.^3^	2023	HAM10000	Optimized ResNet with Whale optimization algorithm for melanoma detection.	Only tested on the HAM10000 dataset.
Keerthana et al.^29^	2022	ISBI 2016	Hybrid CNN models with machine learning for skin cancer classification with an accuracy of 88.02%.	The evaluation is limited to the ISBI 2016 data set.
Xu et al.^30^	2020	ACS Database	soft computing techniques for computer-aided diagnosis of skin cancer with 95% accuracy.	Special focus on soft computing methods.
Sughati et al.^31^	2023	HAM10000	Ensemble model using ResNet50 with focal loss and class weight techniques achieving 93% accuracy.	Focus on handling dataset imbalance.
Popescu et al.^32^	2022	HAM10000	Collective intelligence approach using CNN models, improving accuracy by 3%.	Rely on CNN outputs; no alternative methods investigated.
Albahar^35^	2020	ISIC 2018	Classification of skin lesions using CNN with a novel regularizer that achieves 97.49% accuracy.	Regularization performance in conjunction with specific parameters.
Zhao et al.^39^	2021	ISIC-2019	Classification of dermoscopy images using StyleGAN and DenseNet201, with an accuracy of 93.64%.	Limited to the ISIC-2019 dataset.
Viknesh et al.^40^	2023	ISIC dataset	CNN-based skin lesion classification model achieving 91% accuracy.	Limited by the datasets used (ISIC dataset).
Hoang et al.^54^	2022	HAM10000	Presented a hybrid approach using DenseNet and residual networks, achieving 95% accuracy.	Limited to certain CNN architectures.

## Methods

In this section, we present the experimental methodology of the proposed classification approach for skin cancer in detail. It also describes in detail the data used in the study and its cleaning. The techniques used to improve data accuracy and prepare the data for deep learning analysis are also explained.

### Dataset description

**Fig. 1:**
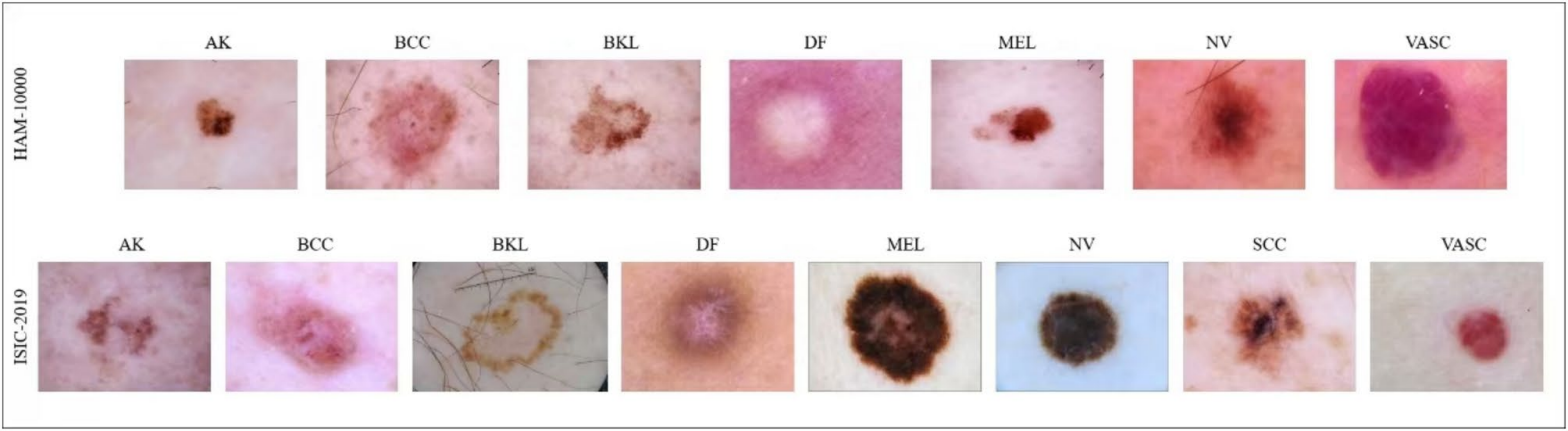
Examples of dermatoscopic images per class from the ISIC-2019 and HAM10000 dataset.

To evaluate the proposed model, we use two dermoscopy image datasets in this study. This section provides information about these datasets. Both the HAM10000 and ISIC-2019 datasets provide real-world examples from dermatology and dermoscopic imaging due to their diversity, clinical challenges, differences in images and respect for ethics. Using these collections, researchers can determine how well ML algorithms can be used to detect real-world dermatological problems and help clinicians. Figure 1 shows the different classes in both datasets.

#### HAM10000 dataset

**Fig. 2:**
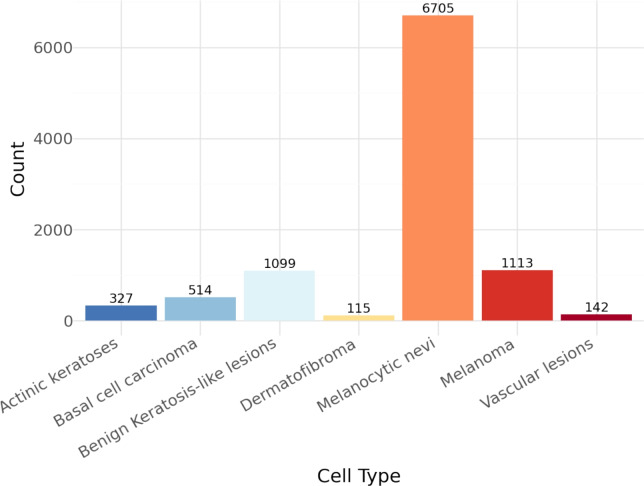
Details of each class contained in the HAM10000 dataset.

Using the widely recognized HAM10000 (Human Against Machine training data for 10,000 images), dermatologists can categorize skin lesions in research. The database contains 10,015 images categorised into seven classes: melanocytic nevi (nv), melanomas (mel), benign keratosis-like lesions (bkl), basal cell carcinomas (bcc), actinic keratoses (akiec), vascular lesions (vasc) and dermatofibromas (df). To make deep learning models accurate, a large selection of high-quality images is required. This dataset collects and curates a large number of images of skin diseases that occur in typical dermatological cases. As the images for this dataset come from many groups of people, they include a variety of skin tones, appearances of dermatological lesions and image quality, making them useful for analyzing classification models. The dataset is not balanced, as some categories have far fewer instances than others. As a result, the machine learning models could make biased assumptions and cause degradation of the classification results. Figure 2 shows that the data is not evenly distributed and illustrates the need for these techniques.

#### ISIC-2019 dataset

**Fig. 3:**
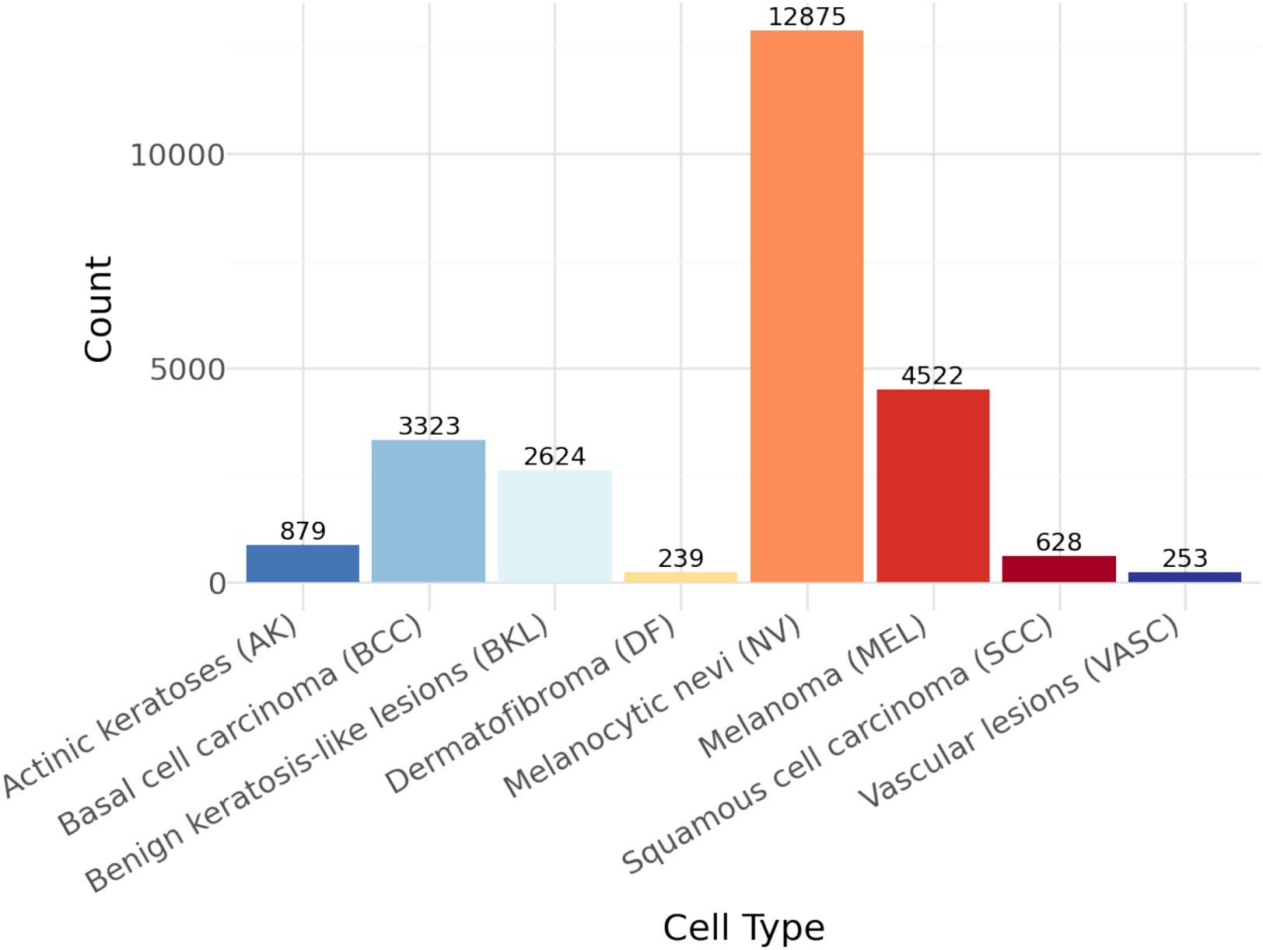
Details of each class contained in the ISIC-2019 dataset.

The ISIC-2019 dataset of the International Skin Imaging Collaboration (ISIC) contains various dermatoscopic images for the classification of skin lesions into nine different categories. The main purpose of this dataset is to develop and test machines capable of recognizing skin features in the images obtained. The dataset contains 25,343 images that have been assigned to eight diagnostic categories for training purposes. Each image is accompanied by a text section describing the type of skin lesion. Thus, the data can be used in developing and testing the performance of deep learning methods in a dermatologic context. ISIC-2019 is one of the most comprehensive publicly available databases in the field of skin disease detection and the large number of different cases makes it a suitable testbed for analyzing dermatological images using algorithms. As the images in the dataset are very diverse and represent multiple imaging situations, the dataset is better suited for clinical practice. Figure 3 illustrates the problem of class imbalance in the ISIC-2019 dataset, where certain classes are significantly underrepresented.

### Preprocessing

Medical images usually have low contrast, noise, artifacts and color distortions. Therefore, pre-processing is necessary to improve their quality and make the models more robust. Pre-processing helps to make the inputs consistent, reduce differences between them and improve the ability of deep learning models to extract important features.

#### Normalization

Normalizing the data helps the system deal with different pixel values due to differences in lighting. Each pixel $I_{(x,y,c)}$ in an image is normalized using:

1 $$\begin{aligned} \tilde{I}_{(x,y,c)} = \frac{I_{(x,y,c)} - \mu _c}{s_c}, \quad \forall x, y, c \end{aligned}$$

where: - $\mu _c$ and $s_c$ are the channel-wise mean and scaling factor, respectively, - Standard values used: $\mu = [0.485, 0.456, 0.406]$, $s = [0.229, 0.224, 0.225]$, - $\tilde{I}$ represents the normalized image.

The process ensures that all images are similar in brightness, which leads to faster changes and fewer annoying changes during training.

#### Resizing

All images for ConvNeXt and ViT models have been resized to 224 $\times$ 224 pixels to make them compatible.

2 $$\begin{aligned} \hat{I} = R(I, h, w) \end{aligned}$$

where $R(\cdot )$ denotes the scaling operation and $h = w = 224$ represents the new height and width. Bilinear interpolation is used to maintain image quality while optimising computational efficiency.

#### Data augmentation

Data augmentation adds artificial changes to the training data to strengthen the generalisation ability of the model and reduce overfitting. The following data augmentation strategies are used:

- Random Flipping:

3 $$\begin{aligned} \hat{I} = {\left\{ \begin{array}{ll} I, & p < 0.5 \\ F(I), & \text {otherwise} \end{array}\right. } \end{aligned}$$

where $p \sim U(0,1)$ is a random probability and $F(I)$ represents horizontal or vertical flipping.

- Random Rotation:

4 $$\begin{aligned} \hat{I} = T(I, \theta ), \quad \theta \sim U(-\theta _{max}, \theta _{max}) \end{aligned}$$

where $T(I, \theta )$ denotes the transformation function that rotates an image by $\theta$ degrees.

- Color Augmentation:

5 $$\begin{aligned} \tilde{I} = \alpha I + \beta \end{aligned}$$

where: - $\alpha \sim U(0.8,1.2)$ controls contrast adjustments, - $\beta \sim U(-0.1,0.1)$ modifies brightness levels.

#### Final preprocessing pipeline

The complete preprocessing transformation applied to an input image $I$ is given by:

6 $$\begin{aligned} I^* = C(T(F(R(N(I))))) \end{aligned}$$

where: - $N(I)$ is normalization, - $R(I)$ is resizing, - $F(I)$ is flipping, - $T(I)$ is rotation, - $C(I)$ is color jittering.

The images are standardized in this pre-processing pipeline, the representation of features is adopted and the model is better adapted to a dermatological condition.

### Data balancing using synthetic minority over-sampling technique (SMOTE)

A major challenge in classification tasks is ensuring fairness and accuracy when working with unbalanced data. In these cases, there are usually only a limited number of dominant sample classes and much fewer minority classes, which are often of great importance or dramatic importance for a diagnosis. Conventional deep learning classifiers, which are optimized for the highest overall accuracy, tend to favor the majority class and reduce the prediction accuracy for the minority classes. To solve this problem, an oversampling strategy is usually used. It consists of increasing the size of the data set by creating synthetic examples or duplicating the existing examples in the underrepresented classes, which improves the class balance and the representation of minority patterns by the model.

The Synthetic Minority Over-sampling Technique (SMOTE) is one of the most robust and common methods used to introduce new samples that are interpolated between the existing minority class samples in this study^55,56^. This method was applied to both the ISIC-2019 and HAM10000 datasets, both of which have significant class imbalance, as can be seen in Figs. 2 and 3 respectively. The use of SMOTE ensures a balanced distribution of all diagnostic classes during training and facilitates learning in all areas.

By incorporating SMOTE into our preprocessing pipeline, we improve the ability of our proposed model, MedFusionNet, to learn discriminative features for all classes. This approach contributes to higher robustness, better generalisation and better performance in dermoscopic image classification.

## MedFusionNet: A ConvNeXt-ViT framework for skin cancer classification

**Fig. 4:**
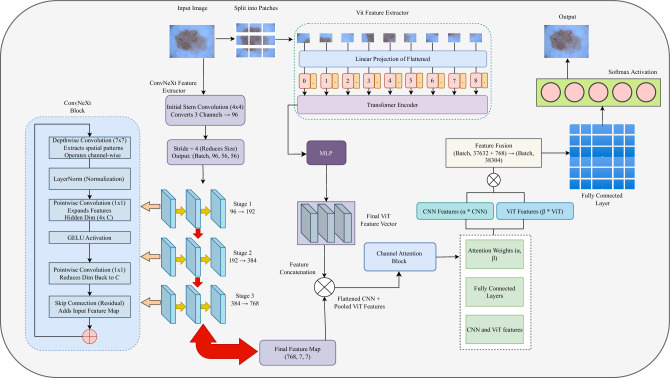
Workflow of the proposed model.

This study aims to develop a deep learning-based classification system, MedFusionNet, for the automatic detection of skin cancer. This framework integrates ConvNeXt for spatial feature extraction and Vision Transformer (ViT) for global feature representation, leveraging the strengths of both CNN- and Transformer-based architectures. Figure 4 provides an overview of the proposed modelling workflow.

In this study, two publicly available datasets, HAM10000 and ISIC-2019, are used to ensure the diversity of lesion types and robustness of classification performance. The input images are resized to 224 $\times$ 224 pixels and normalized to standardize the pixel distributions to speed up training convergence and reduce computational costs.

### Feature extraction via ConvNeXt and vision transformer

The proposed MedFusionNet model consists of two parallel branches of feature extraction: one using ConvNeXt for local feature learning and the other using ViT to capture long-range dependencies.

ConvNeXt feature extraction (CNN-based hierarchical representation) ConvNeXt serves as a CNN-based backbone in which convolution operations extract spatially localized features. A standard convolution operation in ConvNeXt can be expressed as follows:

7 $$\begin{aligned} F_c = \text {ReLU} (W_c \circledast X + B_c), \end{aligned}$$

where $X$ is the input image, $W_c$ is the kernel filter, $B_c$ is the bias and $\circledast$ denotes the convolution operation. The activation function ReLU introduces nonlinearity and thus improves the model’s ability to capture complex patterns.

Each ConvNeXt layer is further refined using convolution in depth and layer normalization (LN) to stabilize the training:

8 $$\begin{aligned} F_{CNN} = LN(Conv_{depthwise}(F_c)). \end{aligned}$$

This formulation ensures an efficient hierarchical feature representation and facilitates effective classification.

Vision Transformer feature extraction (global context learning) ViT processes images as non-overlapping patches of dimension $P \times P$, where each patch is transformed into an adaptive embedding:

9 $$\begin{aligned} E_p = W_p \cdot P_i + B_p, \end{aligned}$$

where $P_i$ is the vectorized patch representation, $W_p$ is the projection matrix and $B_p$ is the bias term. ViT uses multi-head self-attention (MHSA) to capture global feature dependencies, which are calculated as follows:

10 $$\begin{aligned} A(Q, K, V) = \text {softmax} \left( \frac{Q K^\top }{\sqrt{d}} \right) V, \end{aligned}$$

where $Q, K, V$ are query, key and value matrices and $d$ is the scaling factor for normalization. The final ViT feature representation is calculated as follows:

11 $$\begin{aligned} F_{ViT} = \sum _{i=1}^{N} \alpha _i V_i, \end{aligned}$$

where $\alpha _i$ are the self-attention values assigned to each feature vector.

### Attention-based feature fusion mechanism

An adaptive attention-based fusion strategy is used to efficiently merge the feature representations^57^ of ConvNeXt and ViT. For the extracted feature maps $F_{CNN}$ and $F_{ViT}$, the attention weights are assigned dynamically:

12 $$\begin{aligned} W_c= & \sigma (W_f \cdot F_{CNN}), \end{aligned}$$

13 $$\begin{aligned} W_v= & \sigma (W_f \cdot F_{ViT}), \end{aligned}$$

where $W_f$ is a trainable parameter and $\sigma (\cdot )$ denotes the softmax function that ensures a probability-based weighting mechanism. The final merged representation is as follows:

14 $$\begin{aligned} F_{final} = W_c \odot F_{CNN} + W_v \odot F_{ViT}. \end{aligned}$$

where $\odot$ represents the element-wise multiplication that ensures weighted feature contributions.

Compared to conventional fusion methods such as simple concatenation, gating or cross-attention mechanisms, our adaptive, attention-based strategy offers several advantages. Concatenation lacks flexibility and treats all features equally, while gating and cross-attention introduce significant architectural complexity and computational cost. In contrast, our method uses learned softmax-normalised weights to adaptively prioritise ConvNeXt- or ViT-derived features for each input. This enables dynamic modelling of feature relevance without increasing the number of parameters or requiring complex alignment modules. As a result, the model remains lightweight while achieving superior classification performance, especially when handling the diverse visual patterns in dermoscopic images.

### Classification and output prediction

The classification header consists of fully connected layers followed by non-linear activations:

15 $$\begin{aligned} h = \text {ReLU}(W_h \cdot F_{final} + b_h), \end{aligned}$$

where $W_h$ and $b_h$ are the weighting and bias terms. The softmax activation function is applied in the last output layer:

16 $$\begin{aligned} P(y=j | X) = \frac{\exp (h_j)}{\sum _{i=1}^{N} \exp (h_i)}, \end{aligned}$$

where $N$ is the number of classes. The predicted label is then given by :

17 $$\begin{aligned} \hat{y} = \arg \max _j P(y=j | X). \end{aligned}$$

### Training and optimization

The training objective is to minimize the categorical cross-entropy loss:

18 $$\begin{aligned} \mathscr {L} = -\sum _{i=1}^{N} y_i \log P(y=i | X), \end{aligned}$$

where $y_i$ represents the ground truth. The Adam optimizer is used for gradient-based optimization, where the learning rate $\eta _t$ is updated dynamically:

19 $$\begin{aligned} \eta _{t+1} = \eta _t \times \frac{1}{1 + \gamma t}, \end{aligned}$$

where $\gamma$ is the decay factor that controls the adjustment of the learning rate. The model is trained for 100 epochs with a batch size of 16.

**Fig. 5:**
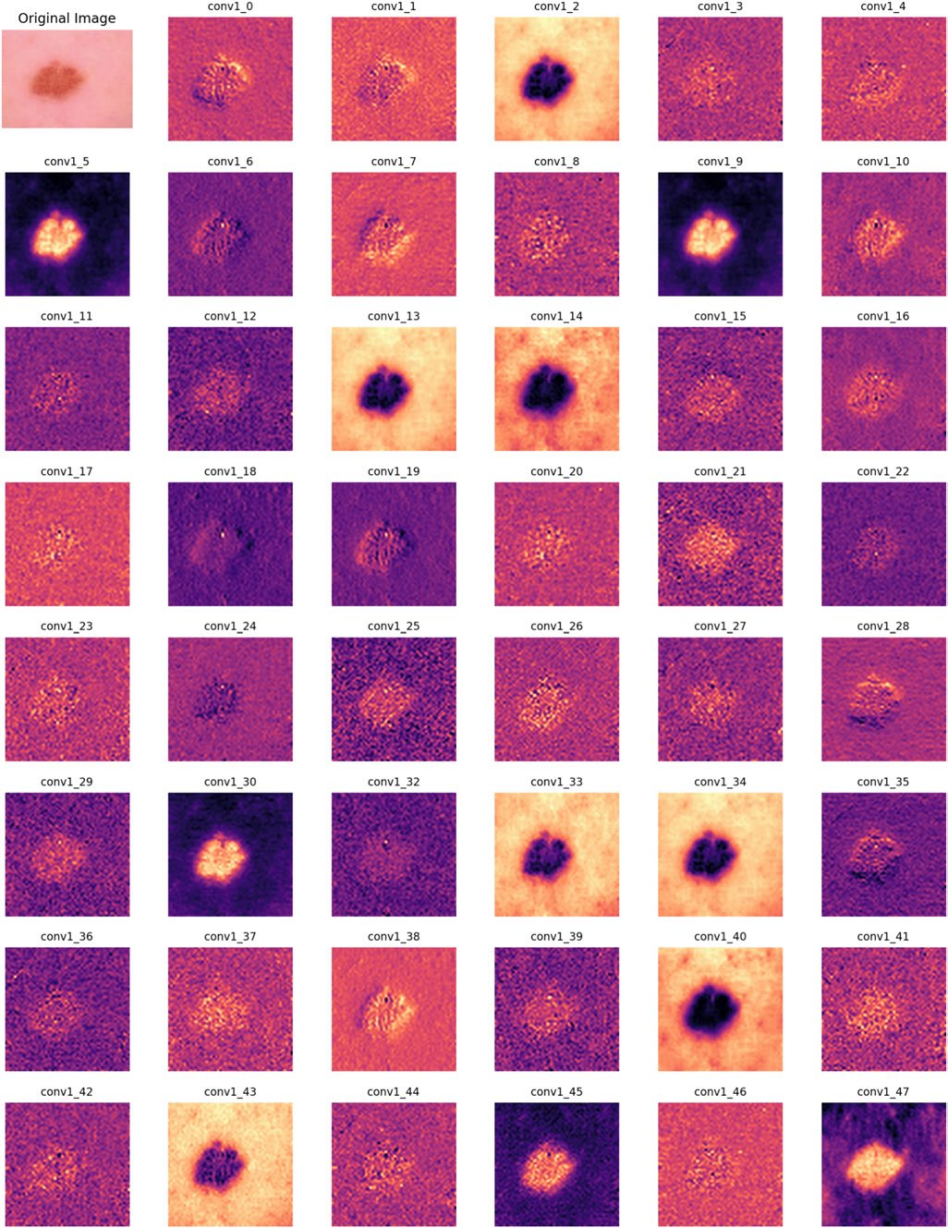
First-layer feature maps of MedFusionNet, showing the original image (top left) and 47 feature maps.

### Model interpretability with Grad-CAM

Figure 5 illustrates the feature extraction behaviour of the first convolutional layer in MedFusionNet. The top-left panel shows the original dermoscopic image, followed by 47 representative feature maps that highlight distinct spatial responses. These activations indicate that the model captures diverse texture and colour patterns related to lesion morphology, providing an interpretable view of its early convolutional processing. To improve transparency, Gradient-weighted Class Activation Mapping (Grad-CAM) is used to generate attention heatmaps that highlight the most influential regions in the image. Grad-CAM calculates the importance of feature maps based on:

20 $$\begin{aligned} A^c_k = \frac{1}{Z} \sum _{i} \sum _{j} \frac{\partial y^c}{\partial F^k_{i,j}}, \end{aligned}$$

where $A^c_k$ represents the contribution of the feature map $k$ to the class $c$ and $Z$ is a normalization factor. The final Grad-CAM heatmap is then calculated as follows:

21 $$\begin{aligned} M^c = \text {ReLU} \left( \sum _k A^c_k F^k \right) . \end{aligned}$$

**Fig. 6:**
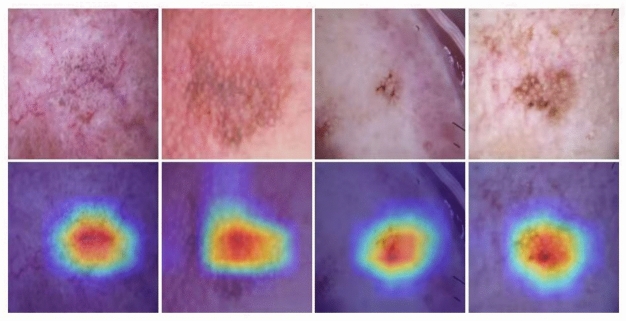
Grad-CAM on random sample test image.

Figure 6 illustrates the Grad-CAM visualisation for randomly selected samples and confirms that MedFusionNet effectively attends to clinically significant lesion regions. While Grad-CAM is a standard qualitative interpretability tool, it should be interpreted with caution, as these visualizations can be sensitive to artefacts from noise or bias. In our study, Grad-CAM is used primarily for descriptive visualization to confirm that MedFusionNet’s attention aligns with clinically relevant lesion areas, rather than as definitive proof of model reasoning.

This study introduces MedFusionNet, a ConvNeXt-ViT-based, attention-driven system for automatic classification of skin cancer. By integrating CNN and Transformer architectures alongside self-attention-based feature fusion, the proposed model achieves improved lesion differentiation and classification accuracy. Experimental results confirm the effectiveness of MedFusionNet in improving interpretability, robustness and real-world clinical applicability for dermatological AI.

**Algorithm 1:**
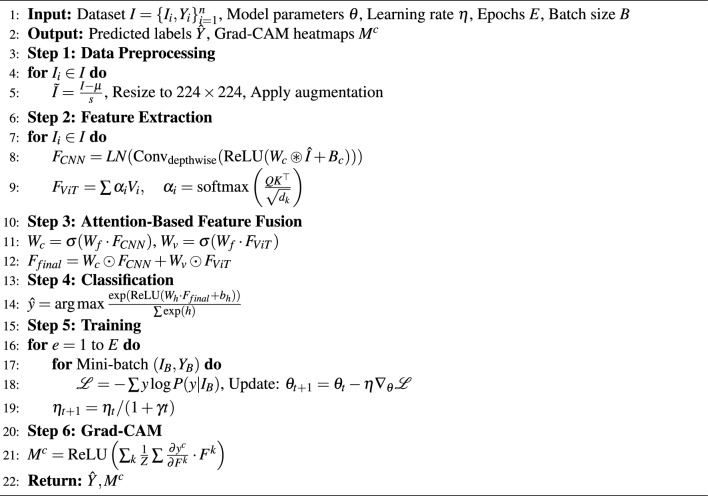
MedFusionNet: ConvNeXt-ViT skin cancer classification.

## Experimental results and discussion

In this section, the performance of MedFusionNet is carefully analysed to measure how well and reliably it performs the classification of skin cancer. Two large dermoscopy datasets, HAM10000 and ISIC-2019, are used to verify the effectiveness of the proposed model. The experimental data show that MedFusionNet is accurate, precise and reliable in terms of skin lesion classification.

### Performance metrics

The following metrics were used to measure MedFusionNet: Accuracy, recall, precision and AUC. They provide information about the sensitivity of the model in detecting skin cancer.

Accuracy: Measures the proportion of correctly classified instances among all predictions. It is computed as:

22 $$\begin{aligned} \text {Accuracy} = \frac{TP + TN}{TP + TN + FP + FN} \end{aligned}$$

Recall (Sensitivity): Represents the model’s ability to identify positive instances among actual positives correctly:

23 $$\begin{aligned} \text {Recall} = \frac{TP}{TP + FN} \end{aligned}$$

Precision: Indicates the reliability of positive predictions, computed as:

24 $$\begin{aligned} \text {Precision} = \frac{TP}{TP + FP} \end{aligned}$$

Specificity (True Negative Rate - TNR): Measures the model’s ability to classify negative instances, computed as: correctly

25 $$\begin{aligned} \text {TNR} = \frac{TN}{TN + FP} \end{aligned}$$

Area under the ROC curve (AUC): This analysis examines how the model identifies the different classifications discussed in the task. To calculate the AUC of our multi-class classification problem, we implemented the one-vs-remainder (OvR) strategy. Given a total number of $C$ classes, the softmax probabilities $\hat{P}_c$ are calculated for each class $c$ and the AUC is calculated for each class individually. The final score is determined by macro-averaging:

26 $$\begin{aligned} \text {AUC}_{\text {macro}} = \frac{1}{C} \sum _{c=1}^{C} \text {AUC}_{\text {OvR}}(c) \end{aligned}$$

These evaluation metrics validate the robustness of MedFusionNet in the automatic classification of skin cancer and ensure high precision, recall and generalisation across multiple datasets.

### Ablation study on HAM10000 and ISIC-2019 datasets

An ablation study was performed using the HAM10000 and ISIC-2019 datasets to evaluate the individual contributions of each component of MedFusionNet. Each data set was kept separate and experiments were performed and the results of accuracy and precision, as well as recall and AUC values, are listed in Table 2. The full MedFusionNet performs best on average, providing an accuracy of 98.80% for HAM10000 and 97.90% for ISIC-2019. When the ViT module is removed, the accuracy drops to 97.10% and 96.30%, respectively, considering its importance for feature extraction from images. Without ConvNeXt, the accuracy drops further to 96.80 for HAM10000 and 95.80 for ISIC-2019. Removing the attention fusion component leads to a drop in performance to 96.50% and 95.50%, respectively, confirming its importance for multi-level feature integration. Turning off data augmentation reduces accuracy drops to 95.90% for HAM10000 and 94.70% for ISIC-2019, highlighting the importance of data diversity in training.

**Table 2:** Ablation Study on HAM10000 and ISIC-2019 Datasets.

Model Variant	HAM10000				ISIC-2019			
	Acc.	Prec.	Rec.	AUC	Acc.	Prec.	Rec.	AUC
MedFusionNet (Proposed)	98.80%	0.95	0.97	0.99	97.90%	0.96	0.98	0.99
MedFusionNet – Without ViT	97.10%	0.94	0.96	0.97	96.30%	0.93	0.95	0.96
MedFusionNet – Without ConvNeXt	96.80%	0.93	0.95	0.96	95.80%	0.92	0.94	0.95
MedFusionNet – Without Attention Fusion	96.50%	0.92	0.94	0.95	95.50%	0.91	0.93	0.94
MedFusionNet – Without Data Augmentation	95.90%	0.90	0.92	0.94	94.70%	0.89	0.91	0.93
MedFusionNet – Concat Fusion	95.65%	0.89	0.91	0.93	94.80%	0.88	0.90	0.92
MedFusionNet – Cross-Attention Fusion	96.80%	0.94	0.96	0.97	96.05%	0.92	0.95	0.96

The study also presents two simpler fusion methods, such as simple feature concatenation and cross-attention. In the case of HAM10000, these approaches achieve an accuracy of 95.65 percent and 96.80% percent respectively, compared to 94.80% percent and 96.05% percent for ISIC-2019. While cross-attention performs better than concatenation, both fall short of the proposed adaptive attention fusion, further emphasising its effectiveness and computational efficiency when combining spatial and contextual features.

### Comparison with transfer learning models

To further validate the performance of MedFusionNet, we compared it with some transfer learning models such as ResNet50, DenseNet121, MobileNetV2 and ViT-B16.Table 3 gives an overview of MedFusionNet with different hyperparameters and shows how the model efficiently categorises dermatological images from the HAM10000 and ISIC-2019 datasets.

Table 3 presents a comparative analysis of MedFusionNet and different transfer learning models based on key hyperparameters such as learning rate, optimizer, batch size and epochs. In addition, the classification accuracy on two benchmark datasets is given: HAM10000 and ISIC-2019. The results clearly show that MedFusionNet with optimal hyperparameters (learning rate of $1e^{-4}$, Adam optimizer, batch size of 16 and 100 epochs) outperforms all other tested models and achieves the highest accuracy of 98.8% on HAM10000 and 97.9% on ISIC-2019. Compared to alternative configurations of MedFusionNet, our optimal setup shows a significant improvement in accuracy, demonstrating the crucial influence of hyperparameter tuning. The table also shows that transfer learning models fall short of MedFusionNet’s performance despite their proven effectiveness. For example, ResNet50 trained with a learning rate of $1e^{-3}$ and an SGD optimizer only achieves an accuracy of 94.5% on HAM10000 and 93.8% on ISIC-2019. DenseNet121 and MobileNetV2 also show a lower accuracy on HAM10000 of 95.6% and 94.9%, respectively, highlighting the robustness of MedFusionNet.

Moreover, ViT-B16, a Vision Transformer-based model trained with a learning rate of $1e^{-4}$ and Adam optimizer, achieves an accuracy of 97.1% on HAM10000 and 96.3% on ISIC-2019. While this shows the potential of Transformer-based architectures, MedFusionNet still achieves better performance, highlighting the effectiveness of the proposed fusion-based approach.

**Table 3:** Performance comparison of MedFusionNet and transfer learning models with different hyperparameters.

Model	Learning Rate	Optimizer	Batch Size	Epochs	HAM10000 Acc (%)	ISIC-2019 Acc (%)
**MedFusionNet (Ours)**	**1e-4**	**Adam**	**16**	**100**	**98.8**	**97.9**
MedFusionNet	1e-3	Adam	16	100	97.5	96.4
MedFusionNet	1e-4	Adamax	16	100	98.0	97.2
ResNet50	1e-3	SGD	32	100	94.5	93.8
DenseNet121	1e-4	Adam	32	100	95.6	94.2
MobileNetV2	1e-3	SGD	32	100	94.9	94.0
ViT-B16	1e-4	Adam	16	100	97.1	96.3

Table 3 solidifies MedFusionNet’s position as a robust model for dermatological image classification. Its ability to outperform traditional CNNs and transformer-based models underlines its effectiveness in extracting and utilising complex features, making it an optimal choice for real-world medical applications.

### Computational performance of the proposed model

**Fig. 7:**
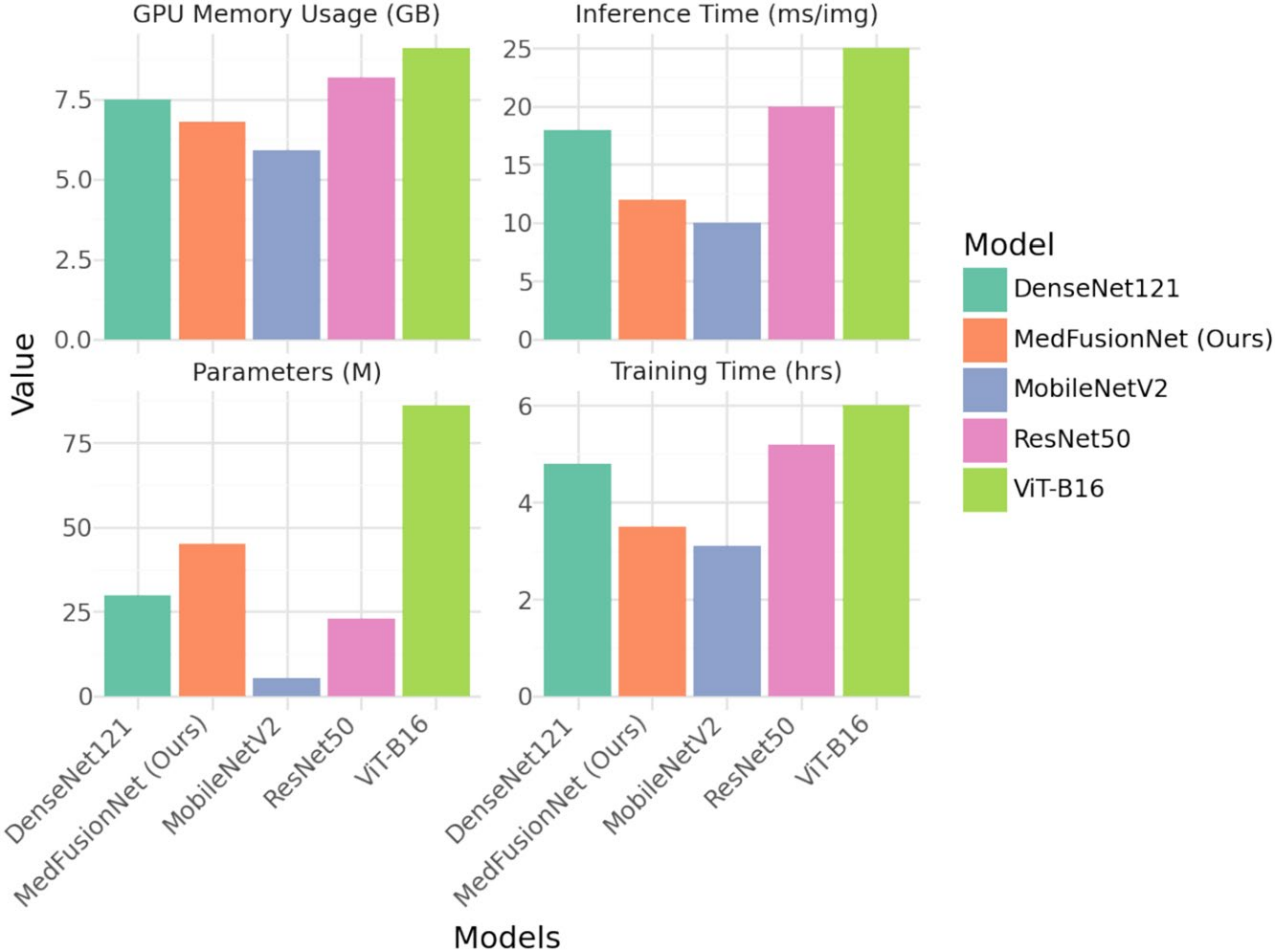
Comparing the computational performance of different deep learning models across multiple metrics.

To evaluate the efficiency of MedFusionNet, we compare it with other models in terms of training time, inference speed, GPU memory consumption and model complexity. As shown in Fig. 7, the results show that MedFusionNet achieves the best trade-off between performance and computational cost.

In addition to the qualitative comparison shown in Fig. 7, absolute computational metrics are provided to assess the feasibility of MedFusionNet for real-time and clinical applications. Table 4 summarizes the floating point operations (FLOPs), average inference time per image, GPU memory utilization and total trainable parameters of MedFusionNet compared with other benchmark models.

**Table 4:** Absolute computational metrics for MedFusionNet and benchmark models.

Model	FLOPs (G)	Inference Time (ms/img)	GPU Memory (GB)	Parameters (M)
**MedFusionNet (Ours)**	**8.6**	**12.1**	6.8	45.2
ResNet50	4.1	20.4	8.2	23.5
DenseNet121	5.7	18.2	7.5	30.1
MobileNetV2	1.8	10.3	**5.9**	**5.3**
ViT-B16	17.3	25.7	9.1	86.4

As shown in Table 4, MedFusionNet achieves a balanced trade-off between computational complexity and accuracy. Although its FLOPs are higher than those of lightweight architectures such as MobileNetV2, its inference time of approximately 12 ms per image indicates suitability for real-time screening on modern GPUs. The efficient attention-based fusion and shared parameter design also help maintain moderate memory consumption compared to pure Transformer models, supporting the model’s practicality in clinical diagnostic settings.

### Performance assessment of MedFusionNet on the HAM10000 dataset

In this subsection, the proposed MedFusionNet model is thoroughly evaluated on the HAM10000 dataset using standard classification metrics and compared with several state-of-the-art deep learning models described in the current literature.

**Fig. 8:**
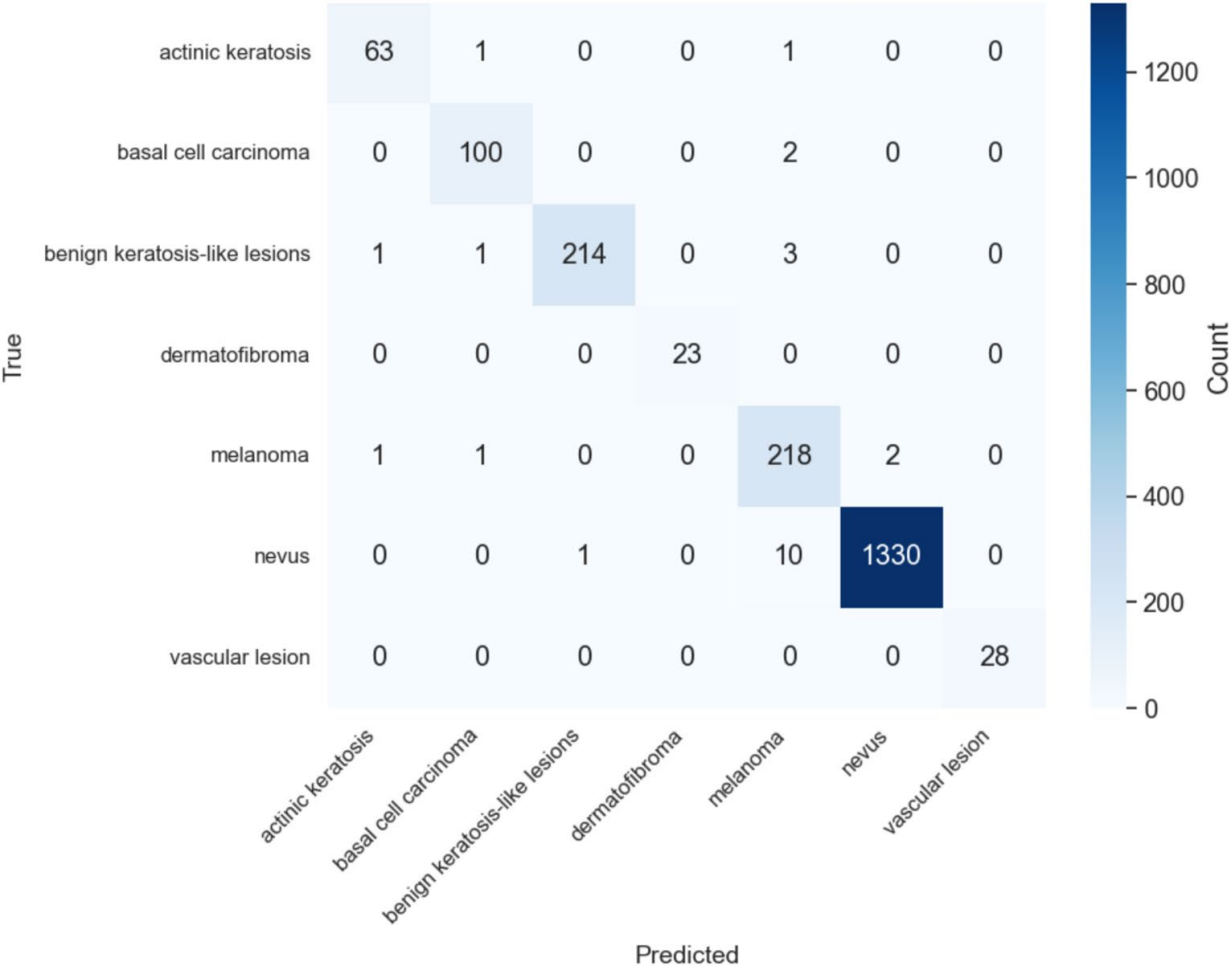
Confusion matrix of MedFusionNet on the HAM10000 dataset.

Figure 8 shows the confusion matrix for MedFusionNet on the HAM10000 dataset and illustrates the excellent class-wise prediction performance. The model accurately classifies major categories such as nevus and melanoma, with nevus achieving 1330 correct predictions from a total of 1341 samples. The melanoma and benign keratosis-like lesion classes also show minimal misclassification. These results reflect the ability of the model to generalise well across a wide range of lesion types.

**Fig. 9:**
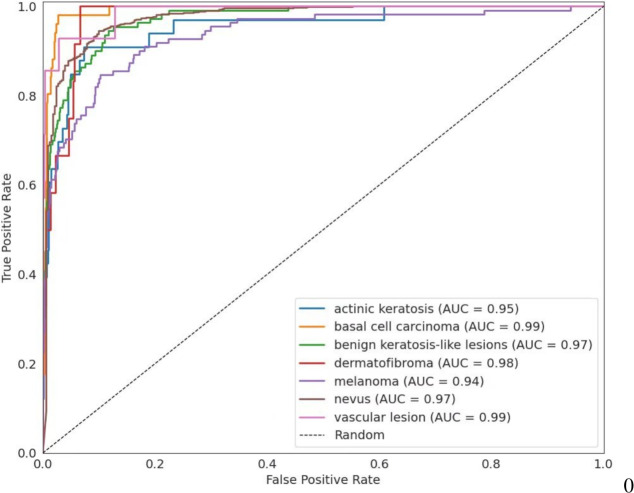
ROC curve for each class on the HAM10000 dataset.

Figure 9 shows the ROC curves for all classes. The nevus class achieves an AUC value of 0.97. Other important classes, such as melanoma and benign keratosis-like lesions, reach AUC values of 0.94 and 0.97, while actinic keratosis and basal cell carcinoma reach 0.95 and 0.99, respectively. These curves illustrate the discriminatory power of MedFusionNet in all lesion categories.

Table 5 provides a comprehensive comparison between MedFusionNet and recent DL-based methods reported in the literature. The studies cover a range of architectures, including classical CNNs, hybrids and transformers.

**Table 5:** Comparison of different models based on accuracy.

Reference	Year	Method	Accuracy (%)
^10^	2019	CNN model	78.00
^58^	2019	Modified MobileNet	83.93
^59^	2019	VGGNet model	85.62
^60^	2019	Modified Inception-v4	94.70
^61^	2020	ResNetXt101	93.20
^23^	2021	CNN model	83.04
^5^	2021	Hybrid AlexNet and SVM	77.80
^62^	2021	ResNet	90.50
^41^	2021	DCNN model	91.93
^63^	2022	Vision Transformer Network (ViT)	94.30
^64^	2022	EfficientNet B4	87.90
^2^	2023	CNN model	82.00
^65^	2023	Modified EfficientNetB3	91.60
^3^	2023	Whale-optimized CNN	92.00
^66^	2023	Hybrid DenseNet and Residual Network	95.00
^67^	2024	deep sequential CNN	96.25
^36^	2024	Deeplabv3+ and vision-based transformer model	100.00
^68^	2025	MobileNet-v2	91.43
**Proposed MedFusionNet**	2025	MedFusionNet	**98.80**

In^66^ a hybrid CNN approach integrating DenseNet and Residual Networks for skin lesion classification was presented. The model achieved an accuracy of 95.00%, which is better than known network models such as InceptionV3, VGG19 and VGG16. In^63^, a Vision Transformer-based model for skin lesion classification was proposed. The transformer architecture enabled the model to capture global dependencies and it achieved a significant accuracy of 94.30%. A deep CNN model proposed in^41^ was optimized for multi-class classification and achieved an accuracy of 91.93% accuracy. Similarly,^65^ used a modified EfficientNetB3 architecture and achieved 91.60%. In^3^, a Whale optimisation algorithm was integrated into a CNN pipeline, resulting in an accuracy of 92.00%. This method showed improved parameter tuning, but still fell short of the proposed model. Other notable works are^62^, where a standard ResNet model achieved 90.50% and^64^, where EfficientNet B4 was used and achieved an accuracy of 87.90%. In^60^, a modified Inception v4 network was proposed, which achieved a competitive accuracy of 94.70%. While high-performing, it does not outperform MedFusionNet. Earlier CNN-based works such as those of^10^ and^23^, only achieved 78.00% and 83.04% accuracy, respectively, due to the limited depth and architectural innovations.

Compared to all these methods, the proposed MedFusionNet model achieves the highest classification accuracy of 98.80%, significantly outperforming the previous benchmarks. The hybrid combination of ConvNeXt and ViT with attention-based fusion enables robust feature extraction and ensures excellent generalisation and prediction performance on different lesion types.

## Performance evaluation of MedFusionNet on the ISIC 2019 dataset

This section presents the classification performance of the proposed MedFusionNet model on the ISIC 2019 dataset. The evaluation is based on generally recognised classification metrics such as accuracy, precision, recall and AUC to provide a comprehensive understanding of the effectiveness of the model.

**Fig. 10:**
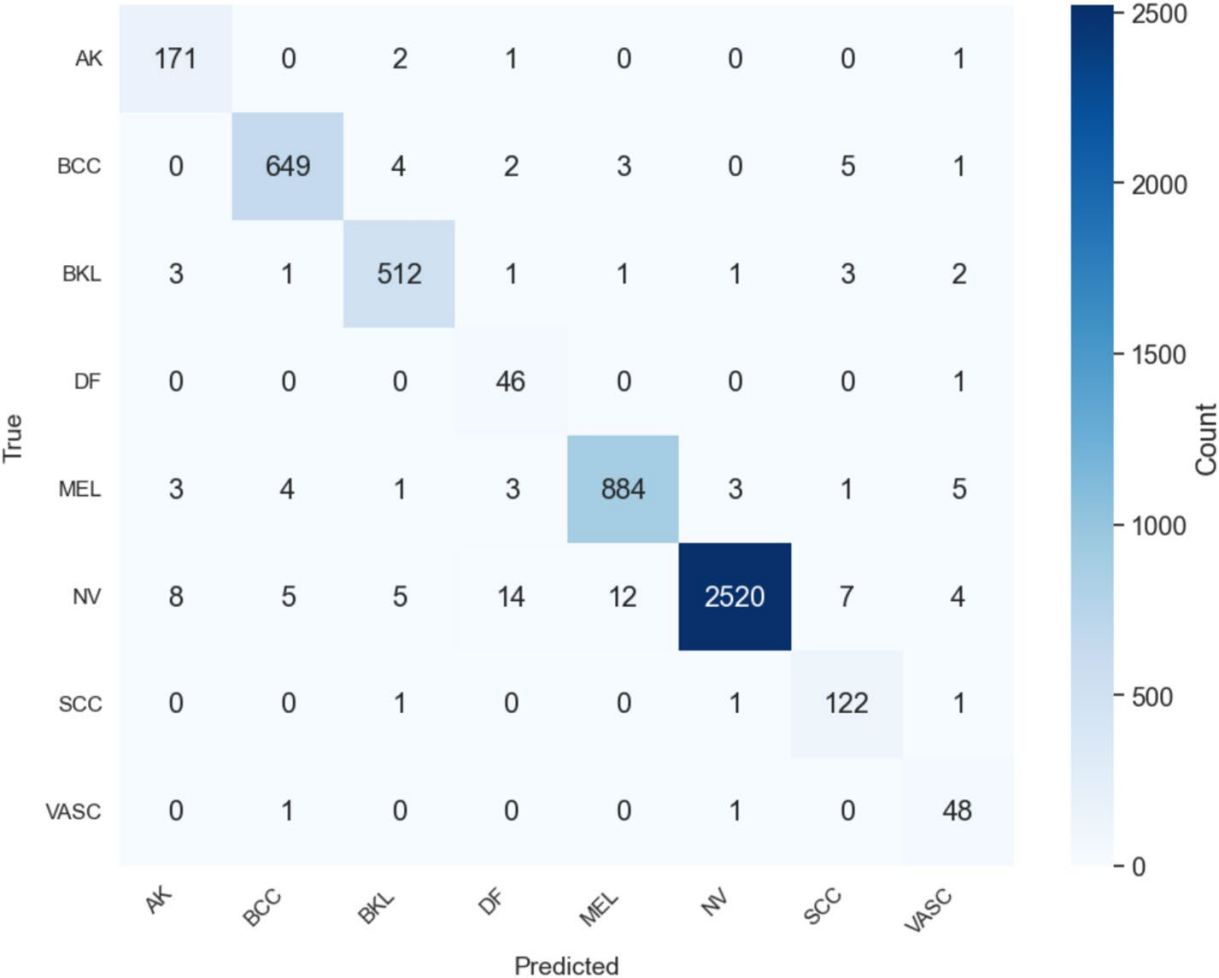
Confusion matrix of MedFusionNet on the ISIC-2019 dataset.

Figure 10 illustrates the confusion matrix obtained from MedFusionNet. The matrix shows a high classification accuracy for all classes. The model achieves significant results for nevus (2520 correct predictions) and melanoma (884 correct predictions), while effectively identifying complex and less represented classes such as dermatofibromas and vascular lesions.

**Fig. 11:**
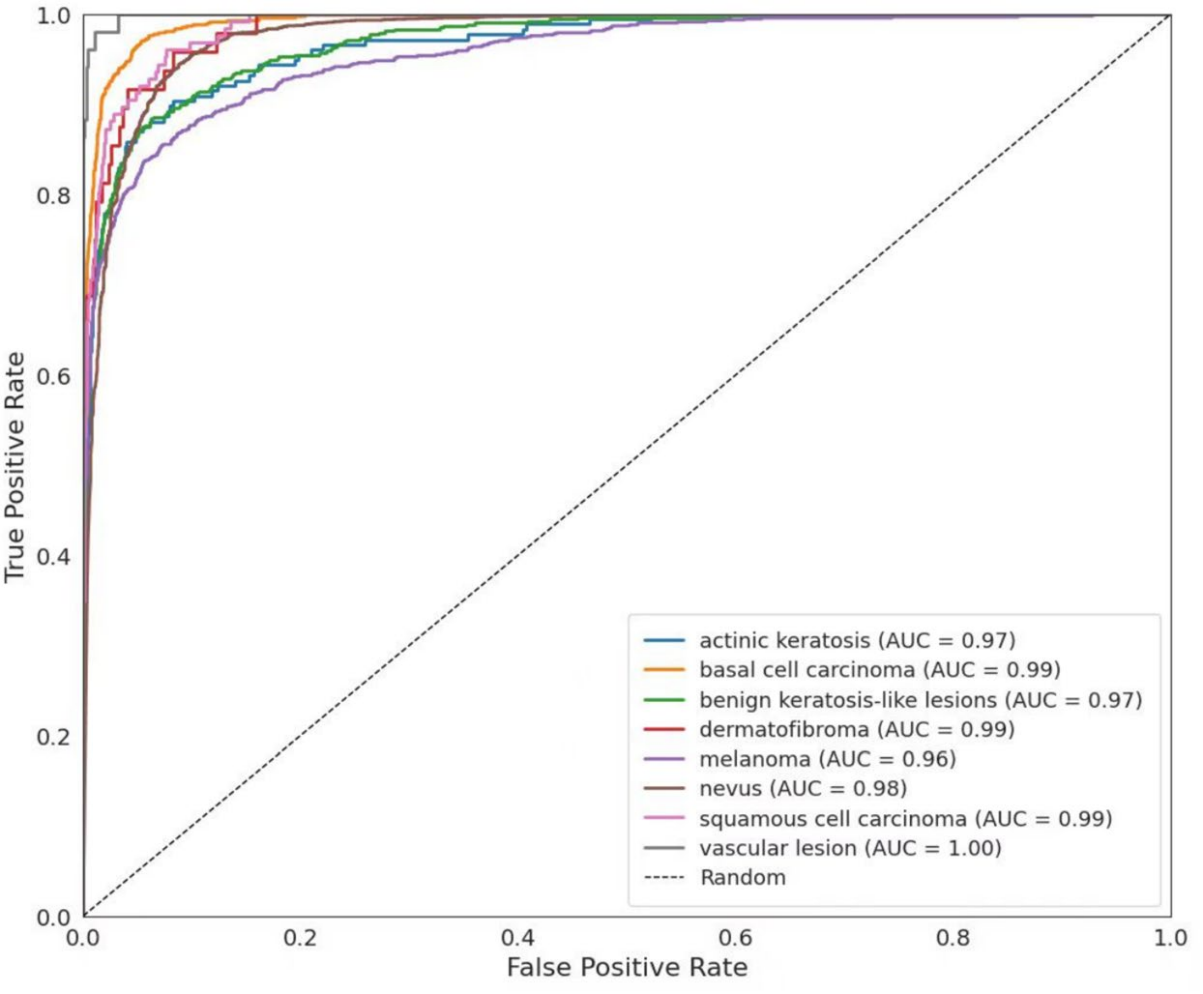
ROC curve for each class on the ISIC-2019 dataset.

The ROC curve in Fig. 11 shows the balance between true positive rate and false positive rate for each lesion class. The AUC values for each class show that MedFusionNet performs particularly well for benign keratosis-like lesions (0.97), dermatofibromas (0.99) and vascular lesions (1.00). Even the lowest-performing class, melanoma, achieved an AUC of 0.96, indicating strong discriminatory power in all categories.

Table 6 shows a comparative analysis of the latest deep learning models evaluated against the ISIC-2019 dataset. The table shows that MedFusionNet achieves the highest accuracy of 97.90%, outperforming all previously reported methods.

**Table 6:** Performance Comparison of Models by Year.

Reference	Year	Method	Accuracy (%)
^21^	2020	CNN model	94.92
^25^	2020	Multi-class SVM (MSVM)	96.25
^23^	2021	CNN model	83.80
^69^	2022	DCNN model	85.00
^33^	2022	CNN model	91.89
^70^	2022	Optimized DenseNet-201	93.00
^71^	2022	ResNet18 model	94.47
^72^	2022	CNN model	96.70
^73^	2022	CNN and VGG16	96.91
^74^	2023	ConvNeXt model	87.37
^75^	2023	VGG19 + SVM	96.00
^76^	2023	Inception-V3	96.40
^77^	2024	CNN-EOSA	97.87
^36^	2024	Deeplabv3+ and vision-based transformer model	97.73
^78^	2025	AlexNet transfer learning	90.90
**Proposed MedFusionNet**	2025	MedFusionNet	**97.90**

In^21^, a traditional CNN-based model was proposed that achieved 94.92% accuracy using standard convolutional blocks. While impressive, it lacked a hybrid feature fusion strategy.^25^ SVM (MSVM) of class was implemented, which achieved 96.25% accuracy by optimizing the decision boundaries for each lesion type. The model in^23^ presented a CNN-based classifier with limited depth, resulting in a lower accuracy of 83.80%. In^69^, a DCNN approach was used, which achieved 85.00% accuracy, but was limited by shallow layers and the lack of global feature modelling. The work in^33^ used a deeper CNN model that achieved a very high accuracy of 91.89%, while^70^ proposed an optimized DenseNet-201 that slightly improved performance to 93.00%. ResNet18 was used in^71^, which led to a robust result of 94.47%. Several hybrid CNNs were introduced in 2022 and 2023.^72^ archived 96.70% using a deep CNN with additional regularization.^73^ fused CNN features with VGG16 and achieved 96.91%. The study in^75^ proposed to combine VGG19 with an SVM classifier and achieved 96.00%. A ConvNeXt-based model was tested in^74^ and achieved 87.37%, while the Inception-V3 architecture in^76^ achieved an accuracy of 96.40%. Table 6, the proposed MedFusionNet outperforms all competing models with an accuracy of 97.90%. Its robust performance can be attributed to its hybrid structure, where ConvNeXt effectively extracts spatial features while ViT captures long-range dependencies. Attention-based fusion helps to give more weight to important features and make dermoscopic images more distinctive. With these architectural improvements, MedFusionNet is ready for clinical use and provides reliable results in the identification of different classes of skin lesions.

## Discussion

In the study, the proposed MedFusionNet system is effective in classifying skin cancer into several categories. The results suggest that an automatic self-learning attention fusion method and the ConvNeXt and ViT branches help in learning details and the overall context introduced by MedFusionNet. With this architecture, the model learns well on dermoscopic images and leads to accurate classification of the image.

MedFusionNet performed well in the HAM10000 assessment and performed particularly well in detecting common classes such as nevus and melanoma. It was seen that the model performed well in generalization, as the AUCs were high and the confusion matrix was low. The model was also effective in the ISIC-2019 classification of dermatofibromas and vascular lesions, although it was similar for other types. MedFusionNet outperformed other SOTA models in terms of accuracy and precision. The approach was better than several established technologies, e.g., traditional CNNs, hybrid CNN-SVM combinations and top-level transformer networks. The outstanding performance was achieved thanks to the attention-based fusion mechanism, which guides the process by context-aware adaptation of the different features. Grad-CAM heatmaps showed that the model paid attention to lesion-specific areas, which improved reliability and explained the model’s predictions.

Despite recent improvements, however, there are some limitations in this area of work. The high computational complexity of such models could limit their use on mobile phones and similar diagnostic devices. And although the model works well on two large datasets, its effectiveness still needs to be verified on clinical datasets from different institutions and with a larger number of patients. It is worth noting that the current design neglects metadata such as age, gender or location of the lesion as potential factors for clearer interpretation and improved diagnosis. However, by monitoring attention, we can better understand the model, but this may also lead to sensitivity to poorly focused or unbalanced data. To overcome these issues in future studies, we could develop lighter versions of the architecture, incorporate different forms of data and analyse the data over time to monitor skin cancer cases. Furthermore, although categorical cross entropy was used as the loss function in this study, exploring more advanced or hybrid loss functions such as focal loss, dice loss or weighted cross entropy could improve performance, especially in the presence of class imbalance.

## Conclusion and future work

In this study, we present MedFusionNet as a flexible deep learning structure that utilises ConvNeXt and ViT via an attention mechanism to classify skin cancer. Using the HAM10000 and ISIC-2019 data, the model achieved better accuracy, precision, recall and AUC than other existing methods. By combining local and global information in this model, it is better able to handle learning from a variety of lesions. By creating Grad-CAM images, the researchers were able to visually explain how the model recognizes lesions in images. Despite the excellent results, MedFusionNet still has some issues. If not enough resources are available or time is short, even complex calculations become a challenge. In addition, the model currently omits information about the patient, such as age, gender and location of the lesion, which could increase the accuracy of the diagnosis. Another limitation is the lack of external validation using different real-world data sets, which is crucial for the assessment of generalisability and clinical reliability. Future work will focus on the developing lightweight versions for mobile and computer use, integrating patient demographic and clinical data for multimodal analysis, evaluating the model with multi-institutional datasets under different imaging conditions and exploring the adaptation of MedFusionNet to diverse imaging modalities, such as breast histology or chest radiography, to assess its generalisability to different diagnostic tasks. We also plan to investigate temporal dermoscopic sequences to support modelling of lesion progression to further increase the clinical relevance of MedFusionNet for the early detection of skin cancer.

## Data Availability

The datasets used in this study are publicly available. The HM10000 dataset can be accessed at https://dataverse.harvard.edu/dataset.xhtml?persistentId=doi:10.7910/DVN/DBW86T and the ISIC 2019 dataset is available at https://challenge.isic-archive.com/data/.
